# Small RNA transcriptome analysis using parallel single-cell small RNA sequencing

**DOI:** 10.1038/s41598-023-34390-7

**Published:** 2023-05-09

**Authors:** Jia Li, Zhirong Zhang, Yinghua Zhuang, Fengchao Wang, Tao Cai

**Affiliations:** 1 National Institute of Biological Sciences, Beijing, China; 2 Department of Thoracic Surgery, Beijing Institute of Respiratory Medicine and Beijing Chao-Yang Hospital, Capital Medical University, Beijing, China; 3 Tsinghua Institute of Multidisciplinary Biomedical Research, Tsinghua University, Beijing, China

**Keywords:** Transcriptomics, RNA sequencing

## Abstract

miRNA and other forms of small RNAs are known to regulate many biological processes. Single-cell small RNA sequencing can be used to profile small RNAs of individual cells; however, limitations of efficiency and scale prevent its widespread application. Here, we developed parallel single-cell small RNA sequencing (PSCSR-seq), which can overcome the limitations of existing methods and enable high-throughput small RNA expression profiling of individual cells. Analysis of PSCSR-seq data indicated that diverse cell types could be identified based on patterns of miRNA expression, and showed that miRNA content in nuclei is informative (for example, cell type marker miRNAs can be detected in isolated nuclei). PSCSR-seq is very sensitive: analysis of only 732 peripheral blood mononuclear cells (PBMCs) detected 774 miRNAs, whereas bulk small RNA analysis would require input RNA from approximately 10^6^ cells to detect as many miRNAs. We identified 42 miRNAs as markers for PBMC subpopulations. Moreover, we analyzed the miRNA profiles of 9,533 cells from lung cancer biopsies, and by dissecting cell subpopulations, we identified potentially diagnostic and therapeutic miRNAs for lung cancers. Our study demonstrates that PSCSR-seq is highly sensitive and reproducible, thus making it an advanced tool for miRNA analysis in cancer and life science research.

## Introduction

miRNAs and other forms of small RNAs are known to regulate many biological processes^1,2^, and miRNA expression is currently used by researchers as a signature of disease diagnosis, prognosis, and the determination of patient responses to treatments^3–5^. However, miRNA expression signatures in tissue biopsies are often masked in data analyses after bulk processing of tissue samples, as these typically contain highly heterogeneous cell types. Thus, highly sensitive and high-throughput single-cell small RNA profiling methods are needed to better explore heterogeneous tissue samples. In contrast to high-throughput methods for single-cell mRNA sequencing^6–9^, which have been successfully applied in various biological and medical research areas, few low-throughput, single-cell small RNA analytical methods have been reported^10–13^.

Small RNA sequencing workflows involve a series of reactions. Briefly, these methodologies first ligate adapters to small RNA molecules using T4 RNA ligase I/II so that the small RNA molecules are flanked by a defined sequence. Next, the ligated small RNA molecules are reverse transcribed into cDNA and amplified by PCR. The existing single-cell small RNA sequencing methods suffer the limitations of low efficiency in that the majority of sequencing reads are from adapter self-ligations (5’ and 3’ adapter dimers) or random error sequences, and the target miRNA read numbers are often low. Additionally, the low-throughput designs of these methods cannot be practically applied for small RNA profiling of highly heterogeneous tissue samples. Here, we developed a method called parallel single-cell small RNA sequencing (PSCSR-seq) to overcome these limitations. We applied PSCSR-seq to analyze the small RNA profiles from cultured cells, and then isolated nuclei and PBMCs for experimental validation. Furthermore, we investigated the small RNA profiles from lung cancer samples.

## Results

### Development of PSCSR-seq

We first explored strategies to improve the efficiency of small RNA library construction. Current small RNA sequencing library preparation strategies require multistep procedures, and the ligation efficiency in the early steps of these methods will strongly affect successful small RNA library construction. In particular, 3' adapter ligation (ligating small RNA to a DNA adapter using modified T4 RNA ligase II) is critical^14^. To increase the ligation efficiency, we used microinjection technology to reduce the volume of the 3' ligation reaction to 1~2 nanolitres. Compared to the usual microlitre-scale reaction, the nanolitre-scale reactions can significantly suppress the formation of side products and improve the target product yield during small RNA library construction (Supplementary Fig. S1). The product yield for small RNA libraries is further improved by a heating step, which likely facilitates the release of small RNA from the RNA-induced silencing complex (RISC). A heating step at 75 °C for 10 min can significantly improve the small RNA library yield compared to that of the control reaction at 25 °C (Supplementary Fig. S2).

Next, we explored strategies to reduce potential experimental biases during small RNA sequencing. In our system, the ligation adapters were carefully designed (see Supplementary Table S3). The ligation adapters contain 8 random nucleotides as a unique molecular index (UMI) that can be used for small RNA counting; this structure can reduce the PCR duplication bias in measurements of small RNA expression. Additionally, random nucleotides close to the ligation junction can help neutralize ligation bias^15–17^. We used high temperatures during the reverse transcription process (carried out by SuperScript III reverse transcriptase) to further reduce the potential formation of side products. Then, two rounds of PCR-based amplification introduce barcodes for labeling cells (round 1) and for multiplexing of samples (round 2) for high-throughput sequencing. Compared with ordinary methods, our sequencing method can decrease the potential biases and more sensitively detect miRNAs (Supplementary Fig. S3).

**Figure 1:**
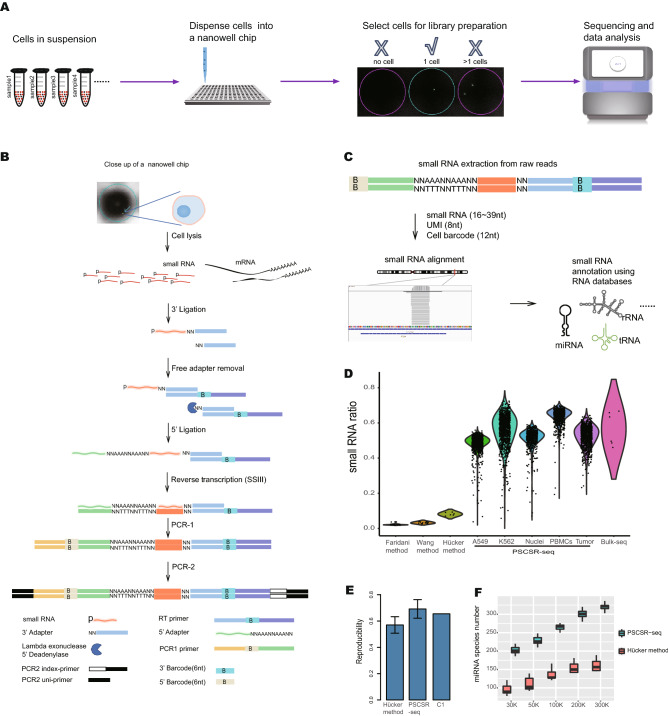
High-quality single-cell small RNA sequencing with PSCSR-seq. (**A**) Schematic of PSCSR-seq. Resuspended cells were dispersed into a nanowell chip. Individual viable cells were selected by a microscope, and small RNA libraries were prepared, followed by sequencing and data analysis. (**B**) Flowchart of single-cell small RNA library preparation. Cells were lysed to release small RNAs. Then, 3' adapters were ligated to these small RNAs, and the remaining 3' adapters were removed. Next, 5' adapters were ligated to these small RNAs. In total, the adapters harbor 8 random nucleotides as a unique molecular index or UMI, which were used for a small RNA counting procedure during data analysis. The ligated small RNAs were then reverse transcribed. In addition, two rounds of PCR-based amplification were used to introduce barcodes for labeling cells (PCR-1) and for multiplexing of samples for high-throughput sequencing (PCR-2) (see the "PSCSR-seq library preparation and sequencing" section of Methods). (**C**) Small RNAs with associated UMIs and cell barcodes were extracted from the raw reads and then aligned to the genome. The small RNA read criteria were as follows: 1, the small RNA length was within 16–39 nt after removal of adapter sequences; and 2, the small RNA sequences needed to be matched to the genome without any mismatches. Small RNAs were further classified into miRNAs or tRNA-derived small RNAs, rRNA-derived small RNAs, as well as other RNA species. The expression level for each class of small RNA was then estimated (see the "PSCSR-seq data analysis" section of Methods). (**D**) Comparison of existing single-cell small RNA sequencing methods. We defined the small RNA ratio as the number of small RNA reads divided by the total number of sequenced reads. The small RNA reads ratios for existing methods were calculated based on published datasets (25 primed human embryonic stem cells were used with the Faridani method^10^, 16 K562 cells were used with the Wang method^11^, 6 A549 cells were used with the Hücker method^13^, and 6 cancer samples with traditional bulk small RNA sequencing method, see the “comparison of methods” section of Methods). (**E**) Reproducibility comparison. The bar plot is based on the average reproducibility values of A549 cells (Hücker method, n = 6), A549 cells randomly chosen from PSCSR-seq results (n = 10), and the reported value from the Fluidigm C1 single-cell mRNA sequencing method^50^. Error bars represent the standard deviation (see the “comparison of methods” section of Methods). (**F**) Boxplot of miRNA numbers per cell detected at different sequencing depths (random downsampling from sequences of A549 cells using the Hücker method and PSCSR-seq, n = 6 and 10 respectively, see the “Comparison of methods” section of Methods).

We further expanded the optimized low-throughput protocol into a highly parallel protocol based on a nanowell (nanolitre-microwell) chip (Fig. 1A,B, see Methods). We compiled an analysis pipeline (Fig. 1C) to decode the small RNA sequences and estimate the small RNA expression of individual cells. Compared with existing methods, PSCSR-seq enables single-cell small RNA profiling for many more cells simultaneously (Fig. 1D). PSCSR-seq was efficient, with well-controlled side products and enhanced small RNA enrichment (Supplementary Fig. S4). PSCSR-seq was highly reproducible across technical replicates, and the cell-to-cell variance of PSCSR-seq was comparable to that of the single-cell mRNA sequencing method (Fig. 1E and Supplementary Fig. S5). PSCSR-seq achieved high sensitivity. We conducted a comparison with the recently reported Hücker method (SBN-CL)^13^. Specifically, using data for A549 cells, PSCSR-seq detected twice as many miRNAs as the Hücker method at the same sequencing depth (Fig. 1F). To explore this new method, we next sequenced 17,565 single cells/nuclei from various biological and clinical samples (Supplementary Table S1).

### Single-cell small RNA transcriptome analysis of cultured cells

**Figure 2:**
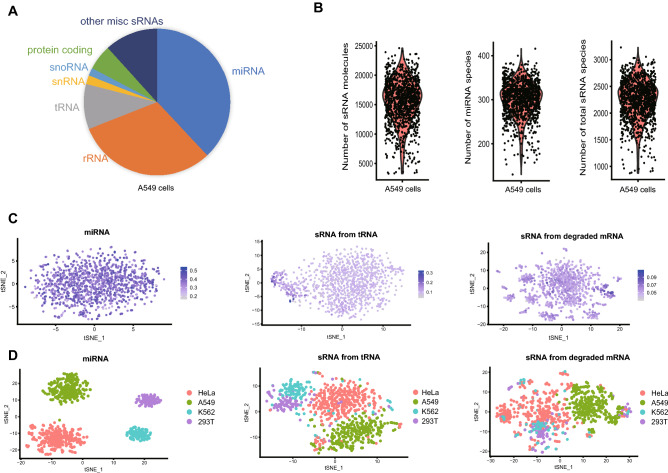
Small RNA profiling of cultured cells via PSCSR-seq. (**A**) Distribution of small RNA molecules in A549 cells. The pie chart shows the proportion of small RNA molecules mapped to each RNA type (also see Supplementary Table S1). (**B**) Violin plots depicting the distribution of total small RNA molecule counts (scored by UMIs) per cell, miRNA species numbers per cell, and the total small RNA species numbers per cell. (**C**) tSNE projection of 1,145 A549 cells calculated from the expression profiles of miRNA and other small RNA forms. The proportion of particular small RNA forms among the total molecules in each cell is overlaid on each tSNE plot. Additionally, see Supplementary Fig. S6F. (**D**) tSNE projection of mixed cells calculated from the expression profiles of miRNA and other small RNA forms. Here, HeLa (n = 356), A549 (n = 242), K562 (n = 120), and HEK293T (n = 109) cells were used for the plots. Additionally, see Supplementary Fig. S6G.

We initially explored the small RNA profiles of A549 cancer cells using PSCSR-seq. We generated 514M raw reads for 1,173 selected cells and after sequencing and data processing, we obtained high-quality data for 1,145 cells (Supplementary Fig. S6A). Each cell had an average of 216K mapped small RNA reads and achieved an estimated ~87% sequencing saturation (Supplementary Fig. S6B). The small RNA complement that can be detected from mammalian cells using PSCSR-seq included miRNAs, small RNAs from rRNAs, tRNAs, snRNAs, snoRNAs, and degraded protein-coding mRNAs, or other miscellaneous small RNAs from unannotated regions. On average, 15,540 small RNA molecules (scored by UMIs) were detected from each cell and miRNAs were of highest abundance (~38% of the small RNA molecules from miRNA loci, Fig. 2A). In the dataset including all cells, PSCSR-seq revealed small RNA species originating from 6,167 genomic loci. The average number of small RNA species in each cell was 2,245. For miRNAs specifically, the whole dataset included miRNA species originating from 1,363 genomic loci, with an average of 301 distinct miRNA species per cell (Fig. 2B).

As expected, our results also confirmed that the expression distribution of the detected miRNA species was highly skewed^18^. A small number of very highly expressed miRNAs were predominant, with an average of only 10 of the most abundant miRNAs accounting for more than half of the overall expressed miRNAs in a given cell (Supplementary Fig. S6C). As we can obtain data from many cells, PSCSR-seq enables precise characterization of the small RNA complement present in a "typical cell" from an A549 cell line, in contrast with what was possible using other low throughput methods. We observed that the miRNA profiles showed considerable homogeneity based on t-distributed stochastic neighbor embedding (tSNE) plots (Fig. 2C). Next, we analyzed four different cell lines (A549, K562, HeLa, and HEK293T) using PSCSR-seq. We found that miRNA profiles could distinguish these cell types when they were mixed, while other forms of small RNAs hardly separated these types of cells (Fig. 2D).

Single-cell analysis revealed multiple cell type marker miRNAs, which significantly overlapped with the bulk sequencing analysis results (Supplementary Fig. S6D). We further compared our data with the expression profiles within a large miRNA atlas with data from 172 different sample types^19^. We found that the average miRNA profiles of individual A549, HEK293T, and HeLa cells were highly consistent with known profiles. That is, the profiles from PSCSR-seq data were most similar to the corresponding profiles in the atlas (Supplementary Table S2). The expression data of K562 cells (a human immortalized myelogenous leukemia cell line) was not present in the existing miRNA atlas database, but a cell ontology analysis based on our PSCSR-seq data indicated that these miRNA profiles were most likely from “leukocytes” or “haematopoietic_cells” (Supplementary Fig. S6E). Taken together, these results emphasized that PSCSR-seq could accurately reveal the miRNA profiles of individual cells.

### Single-cell small RNA transcriptome comparison between nuclei and whole cells

**Figure 3:**
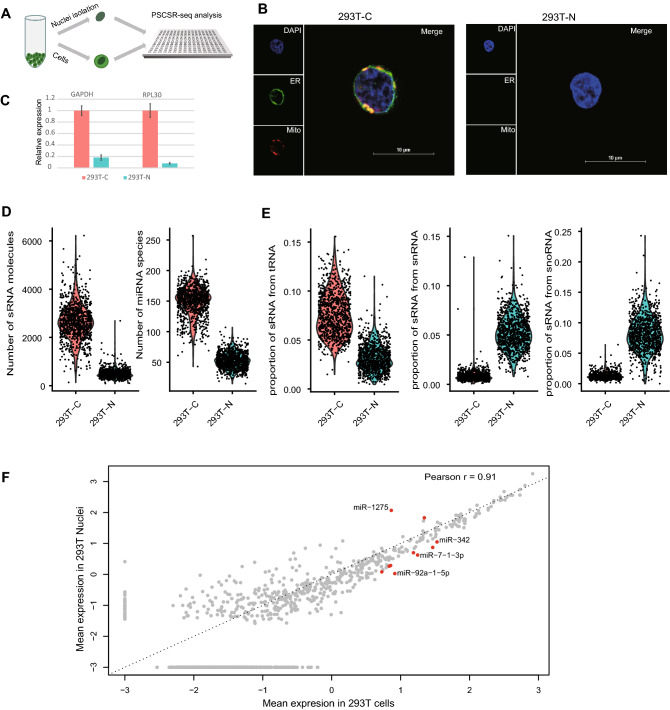
Small RNA profiling of nuclei isolated from HEK293T cells using PSCSR-seq. (**A**) Flowchart of parallel small RNA profiling of nuclei (293 T-N) and whole cells (293 T-C). (**B**) Microscope visualization of nuclei (293 T-N) and whole cells (293 T-C). The samples were stained with DAPI, ER-tracker, and Mito-tracker (see the “nuclei isolation” section of Methods). (**C**) Relative expression levels of marker genes in cells and nuclei. Error bars represent the standard deviation (n = 3). (**D**) Violin plots for comparing the distribution of total small RNA molecule counts per cell, miRNA species numbers per cell, and (**E**) proportions of different forms of small RNAs between nuclei and whole cells (also see Supplementary Table S1). (**F**) Consistency of miRNA expression between nuclei and whole cells. Nuclei or whole cells in PSCSR-seq were pooled together, and the miRNA abundance was averaged. The axis is the log10 transformed. Red dots indicate the differentially expressed miRNAs. For source data, see Supplementary Table S5.

Although single-nucleus analysis strategies are widely used in mRNA expression^20,21^ or DNA modification analysis^22^, single-nucleus small RNA analysis has not yet been explored in the literature. Therefore, we isolated nuclei from human HEK293T cells and applied PSCSR-seq to isolated nuclei. In these experiments, the separation of nuclei and whole cells was verified by RT-qPCR and microscopy experiment (Fig. 3A-C). We used PSCSR-seq to generate the small RNA expression profiles of nuclei (n = 1012) or whole cells (n = 941). On average, the number of small RNA molecules detected per nucleus was ~463, representing 18% of that from whole cells (Fig. 3D). The majority of small RNAs captured were located within 21-24nt (Supplementary Fig. S7A). We observed miRNAs and other diverse forms of small RNAs in nuclei. But the proportions of small RNA forms were significantly different between nuclei and whole cells (Supplementary Table S1). For example, tRNA-derived small RNAs were significantly depleted, but snRNA/snoRNA-derived small RNAs were significantly enriched in nuclei (Fig. 3E). This result was consistent with the knowledge that tRNAs primarily function in the cytoplasm, and snRNA/snoRNAs primarily function in the nucleus.

In nuclei, the patterns of reads mapping to miRNA loci were consistent with the annotations (Supplementary Fig. S7B,C). As expected, fewer miRNA species were detected in nuclei than in whole cells (52 per nucleus vs. 153 per cell). When pooling all the data together, 535 miRNAs were detected in nuclei and 1067 miRNAs in whole cells, with 501 miRNAs sharing overlap between the sample types. The pooled miRNA expression profiles were generally consistent between nuclei and whole cells with exceptions (Fig. 3F. Pearson correlation coefficient r = 0.91). An expression comparison using Wilcoxon rank tests indicated that 10 miRNAs (2% of the total detected miRNAs) were differentially enriched in the nuclei vs. the whole cell samples (see Supplementary Table S5). These miRNAs were relatively less abundant in nuclei except miR-1275 and let-7b. Importantly, our results showed that cell type marker miRNAs were detected in both nuclei and cells. This indicates that nuclei contain informative miRNAs for the characterization of cellular diversity.

We also examined HeLa cells (Supplementary Fig. S8A, B). We isolated the nuclei from HeLa cells and compared the miRNA profiles of 1334 nuclei and 891 whole cells. There is a total of 676 miRNAs detected in nuclei and 1028 miRNAs detected in whole cells (76 miRNAs per nucleus vs. 167 miRNAs per cell, Supplementary Fig. S8C), and the pooled expression profiles between nuclei and whole cells were highly correlated (r = 0.93; Supplementary Fig. S8D). Similarly, all marker miRNAs of HeLa cells can be detected in nuclei. In conclusion, our experiments show that sampling nuclei yields informative data profiles for miRNA expression.

### Single-cell small RNA transcriptome analysis of PBMCs

**Figure 4:**
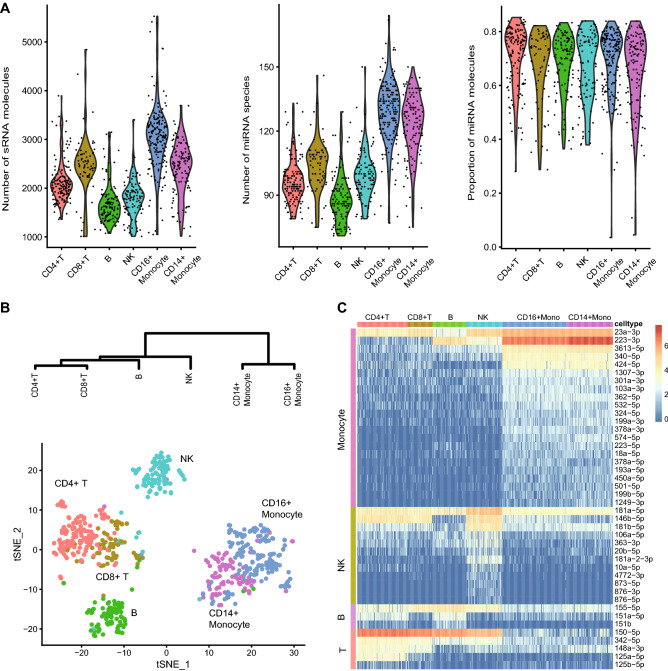
Small RNA profiling of PMBCs using PSCSR-seq. (**A**) Violin plots showing the distribution of small RNA molecule counts, miRNA species numbers, and proportions of miRNA for individual cell populations. (**B**) tSNE plot and hierarchical clustering tree of PBMCs derived from miRNA expression profiles. For the hierarchical clustering analysis, cells of PBMC subpopulations were pooled together, and the miRNA expression was averaged. (**C**) Heatmap presentation of upregulated miRNAs in PBMC subpopulations. Each row indicates particular upregulated miRNA in PBMC subpopulations (highly similar CD4 + /CD8 + T cells and CD14 + /CD16 + monocytes were grouped together). The color bar indicates the miRNA expression level.

PBMCs are small cells known to have low RNA content (~1 pg RNA per cell in PBMCs versus > 10 pg RNA per cell for typical cancer cells)^8^. Comprehensive miRNA profiling in the expression atlas database revealed global hematopoietic markers^18,19^, but high-resolution miRNA markers for subpopulations of PBMCs were lacking. We generated small RNA profiles for the individual components of PBMCs, consisting of 182 CD16+ monocytes, 122 CD14+ monocytes, 146 CD4+ T cells, 66 CD8+ T cells, 116 CD19+ B cells, and 100 CD56+ natural killer (NK) cells (a total of 732 PBMCs). As anticipated, our dataset confirmed that the PBMCs contained many fewer RNA molecules than A549 cells. On average, the number of small RNA molecules detected per cell was ~2,676. miRNA was the major form of the detected small RNA species. An average of 69% of the detected small RNA molecules were miRNAs, and on average, the number of miRNA species per cell was 105, but monocytes had a relatively higher number of miRNAs, with an average of 128 miRNAs per monocyte (Fig. 4A).

As a whole, PSCSR-seq in PBMCs detected a total of 774 miRNA species. The results were comparable to the number of miRNA species identified in a published study using a standard bulk sequencing method (Fantom database, 639–731 miRNAs in PBMCs, approximately 1 µg or 10^6^ cells worth of input RNA for bulk small RNA analysis)^18^. The miRNA profiles of PSCSR-seq were highly consistent with these bulk analysis results. A comparison of our profiling data with published expression profiles from 121 human primary cell types (Fantom database)^18^ revealed that our PSCSR-seq results were most similar to the samples of PBMCs (Supplementary Table S2). Thus, PSCSR-seq was sufficiently accurate to reveal small RNA profiles in PBMCs despite their low RNA content.

A tSNE plot of this data presented distinguishable patterns of miRNA expression profiles for PBMC subpopulations (Fig. 4B). Based on miRNA profiles, approximately 94% of PBMC cell types were classified correctly. When we merged highly similar CD4+/CD8+ T cells and CD14+/CD16+ monocytes, more than 98% of the cells were correctly classified (see the “unsupervised clustering method” section of Methods). A hierarchal clustering analysis based on miRNA profiles separated different subpopulations of PBMCs, which could reflect the cell lineages of PBMCs (Fig. 4B). Lymphocytes diverged from monocytes during hematopoiesis progression, and among lymphoid subpopulations, NK cells diverged from those of B and T cells. A statistical comparison of miRNA expression between NK and T cells showed 1.5 more differentially expressed miRNAs than a comparison between B and T cells. This observation was different from an mRNA profiling analysis, in which NK cells were highly similar to T cells (especially CD8+ T cells)^23^. mRNA profiles only partially correlate with cells’ lineage history^24^, so miRNA readouts provide additional information for lineage analysis.

Our results identified multiple miRNA markers in PBMCs (Fig. 4C). miR-223 and miR-150 have been previously reported to be miRNA markers of hematopoietic cells^19^, and our data confirmed this observation and revealed exclusive expression patterns of miR-223 and miR-150 in myeloid and lymphoid cells. We next tested whether we could use PSCSR-seq to dissect the subpopulations of PBMCs without cell sorting. We therefore applied PSCSR-seq to a fresh PBMC sample from a healthy donor. We validated the different miRNA patterns in these fresh PBMCs and classified the cells into subpopulations. Based on our identified markers, the expression of miR-223 and miR-150 classified PBMCs into myeloid cells and lymphoid cells, and miR-181b or miR-873 could separate NK cells from other lymphocytes. Additionally, miR151a/b could be used to separate B cells from T cells (Supplementary Fig. S9). These results provide a proof-of-concept for the application of PSCSR-seq using highly heterogeneous samples, such as cancer biopsies.

### Single-cell small RNA transcriptome analysis of lung adenocarcinomas (LUADs)

**Figure 5:**
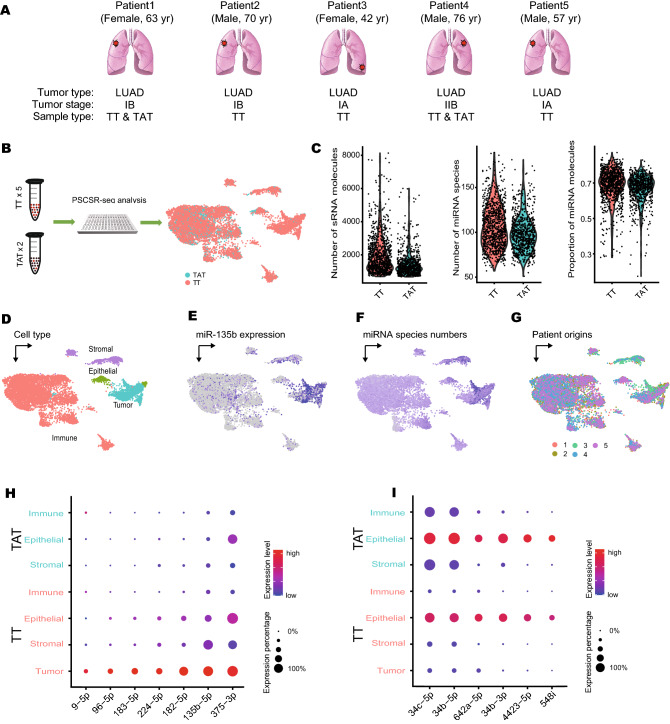
miRNA analysis of lung adenocarcinoma via PSCSR-seq. (**A**) Illustration of sample origins (lung adenocarcinoma, LUAD) and (**B**) PSCSR-seq analysis for cells from tumor tissues (TT) and tumor-adjacent tissues (TAT). (**C**) Violin plots showing the distribution of small RNA molecule counts, miRNA species numbers, and proportions of miRNA for cells from matched TT and TAT. (**D**) miRNA expression profile projection plots indicating different cell subpopulations, (**E**) expression of miR-135b, (**F**) miRNA species numbers, and (**G**) patient origins. (**H**, **I**) Dot plot visualization of tumor-specific differentially regulated miRNAs in the tumor microenvironment. Cell types from TT and TAT are listed on the y-axis. Upregulated miRNAs (**H**) and downregulated miRNAs (**I**) are listed on the x-axis. Dot colors reflect the average expression level (scaled across cell types), and dot sizes indicate the expression percentage in each cell type.

We next explored the application of PSCSR-seq to an analysis of clinical specimens. To do this, we harvested cells from tumor tissues (TT) and tumor adjacent tissues (TAT) collected from 5 lung cancer patients (Fig. 5A), and used PSCSR-seq to analyze a total of 9,533 cells. This included 8,198 (86.0%) cells from TT samples and 1,335 (14.0%) cells from TAT samples (Fig. 5B). The demographic information of the patients is presented in Supplementary Table S4. We detected a total of 1,463 miRNA species (an average of 98 miRNAs per cell). miRNAs again represented the major form among the detected small RNAs, and in fact, an average of 70% of the detected small RNA molecules were miRNAs. The distributions of small RNA molecules, including both UMI count data and miRNA species number per cell, were matched between TT and TAT for this dataset, but the distributions in the TT group had long tails (Fig. 5C). tSNE plots highlighted the different miRNA expression patterns between TT and TAT samples (Supplementary Fig. S10A).

Using a “canonical correlation analysis” method^25^, we integrated all the cells from different patients together and then classified them into cell subpopulations (Fig. 5D,G). We first distinguished tumor cells from non-tumor cells using the expression of miR-135b (Fig. 5E), which is known to be highly expressed in lung cancer^26^. Tumor cells contained a relatively larger number of miRNA species than other normal cells (Fig. 5F). The majority of the annotated tumor cells came from TT samples rather than TAT samples (n = 1,640, 98.8% from TT and 1.2% from TAT). Based on our established miRNA markers and an existing miRNA atlas expression database^18^, we could annotate non-tumor cell populations: immune cells (n = 6,693; 82.5% from TT and 17.5% from TAT), stromal cells (fibroblast or endothelial cells; n = 800, 93.6% from TT and 6.4% from TAT), and normal epithelial cells (n = 400, 76.0% from TT and 24.0% from TAT). An interactive presentation of the annotated data for the lung cancer cells can be found at https://biocaitao.github.io/lungcancer/index.html.

Comparison of tumor cells and non-tumor cells revealed multiple tumor-specific differentially regulated miRNAs. The upregulated miRNAs (Fig. 5H) have been previously reported to function in the tumor inflammatory response, cell proliferation, invasion, and metastasis^26,27^. Additionally, the upregulated miRNAs, including miR-9^28^, 96^29^, 182^30^, and 183^30^, have been reported as serum markers for early lung cancer diagnosis. The known tumor suppressor miRNAs (Fig. 5I) were among the downregulated miRNAs in our dataset (for example, miR-34b/c^31^, 642a^32^, 4423^33^, and 548i^34^). This was attractive as miR-34 family miRNAs are potentially therapeutic miRNAs^35^. With single-base and single-cell resolution, our data showed the different expression patterns of miR-34 family miRNAs in lung adenocarcinomas (Supplementary Fig. S10B). Compared to miR-34a, we observed that miR-34b/c was strongly suppressed in tumor cells. These findings were consistent with the observations of hypermethylation in the promoter region of miR-34b/c in lung cancer samples^36,37^. Taken together, our results confirmed that PSCSR-seq could informatively dissect cell populations and reveal tumor-associated miRNAs from clinical specimens from cancer patients.

## Discussion

Here, we developed a parallel single-cell small RNA sequencing method and demonstrated its application using diverse biological and clinical samples. Our results showed that PSCSR-seq was highly sensitive and reproducible, and possessed superior performance over existing methods in several areas. First, PSCSR-seq greatly alleviated the known issues associated with single-cell small RNA sequencing, such as the high proportion of self-ligation dimmers and the low proportion of miRNA reads. Second, PSCSR-seq is an efficient system. Its adapters were carefully designed to ensure higher ligation efficiency and lower formation of unwanted side products relative to other adapters. Extra reagents such as PEG or ribosomal RNA-masking oligos were not needed in this system. Finally, PSCSR-seq was performed at a nanoliter scale and based on a high-throughput sample management system. Nanoliter scale reactions could increase the concentration of the reactants and reduce the cost of reagents (for PSCSR-seq library construction, the cost was ~$1.6 per cell). Also, all of the analyses in a nanowell chip were performed under the same reaction conditions, the reaction variations could be reduced. Currently, we use the commercially available Takara ICELL8 system, but we expect the protocol can be adapted by other platforms than can operate multi-step nanoliter reactions. PSCSR-seq chose the strict parameters in the analysis pipeline (for example, allowing no mismatches in the small RNA alignment); these settings could facilitate the quantification of miRNA isoforms, some of which only have single-base differences. However, the current pipeline may miss miRNAs with RNA editing, and further improvements to include these miRNAs are needed.

Nuclei contain miRNAs and their unprocessed precursors^38,39^. By analyzing single-nucleus small RNA profiles, our results show that the miRNAs from nuclei were highly informative. The miRNA expression profiles in nuclei were generally consistent with those from whole cells. In single-cell studies, analyzing nuclei is a useful strategy for archived clinical materials or hard to dissociate tissues (for example, brain tissue^21^ or plant tissue^40^). PSCSR-seq paves the way for the small RNA analysis in these samples. Although there is a relatively small number of miRNAs encoded in the genome, single-cell miRNA profiles can be used to infer cell types. We demonstrate that PSCSR-seq can dissect cell populations in lung cancer, and identify tumor-specific miRNAs that are of diagnostic or therapeutic value. Finding suitable miRNA markers or targets in miRNA translational applications has been challenging^35^. Heterogeneous cell compositions largely confound traditional bulk miRNA analysis. PSCSR-seq can provide an advanced tool for miRNA translational studies.

Recent published “polyadenylation and template switching” method (Smart-seq-total) can investigate a broad spectrum of coding and non-coding RNA (including miRNAs) from a single cell^41^. However, the miRNA proportion in the method is low. PSCSR-seq is highly sensitive for miRNA profiling. Given that miRNAs are essential regulators in many biological processes and diseases, PSCSR-seq will have broad applications.

## Methods

### Oligonucleotides design and synthesis

3' adapter (RA3-A2N) was obtained from Takara Biomedical Technology (Beijing, China); 5' adapter (SR5F) was obtained from Sangon Biotech (Shanghai, China). Other oligonucleotides were obtained from Sangon Biotech. All oligonucleotides used in this study are described in Supplementary Table S3.

### Cell culture

A549 cells were cultured in DMEM/F-12 (11320082, Gibco, Thermo Fisher Scientific). HeLa, 3T3, and HEK293T cells were cultured in basic DMEM (C11965500BT, Gibco). K562 cells were cultured in basic RPMI-1640 media (C22400500BT, Gibco). All the cultured media were supplemented with 10% (v/v) fetal bovine serum (26140079, Gibco) and 1% penicillin-streptomycin (15140122, Gibco). The cells were cultured at 37°C in a 5% CO2 humidified incubator. Fresh cells were resuspended with 1 x Dulbecco's phosphate-buffered saline (DPBS, C14190500BT, Thermo Fisher Scientific), and stained with 4',6'-diamidino-2-phenylindole (D1306, Thermo Fisher Scientific) to indicate dead cells. Living cells were sorted using a BD FACSAria III instrument at the facility (National Institute of Biological Sciences, Beijing).

### PBMCs preparation

Cryopreserved CD3+ Pan T cells (PB009-1F-C-5M), CD19+ B cells (PB010-P-F-C), CD14+ monocytes (PB-011-P-F-1-C), and CD56+ natural killer cells (PB012-P-F-C) were purchased from AllCells (Shanghai, China). The CD3+ Pan T cells were stained with APC/Cyanine7 anti-human CD3 Antibody (300425, BioLegend), FITC anti-human CD4 Antibody (300506, BioLegend) and PE anti-human CD8 Antibody (344705, BioLegend) following the manufacturer’s instructions. Then, the Pan T cells were sorted into CD4+ T cells and CD8+ T cells with a BD FACSAria III instrument. CD14+ monocytes were stained with PE anti-human CD14 Antibody (301805, BioLegend) and APC anti-human CD16 Antibody (302011, BioLegend) following the manufacturer’s instructions. CD14+ monocytes were further separated into CD16+ monocytes and CD14+ monocytes (CD16-) with a BD FACSAria III instrument.

For fresh PBMCs, venous blood was collected from a healthy donor into a plastic blood tube spray-coated with K_2_EDTA (367863, Becton Dickinson, NJ, USA). Blood was dissolved in an equal volume of 1 x DPBS solution and added to a SepMate™-15 tube (86415, StemCell Technologies, Canada) containing Histopaque-1077 (10771-100 ml, Sigma-Aldrich, MO, USA). After centrifugation (1200 x g, 10 min), PBMCs were collected, washed twice, and resuspended with a 1 x DPBS solution.

### Nuclei isolation

Cultured HeLa and HEK293T nuclei were isolated according to the “L&W” protocol^42^. All preparations were performed on ice. Briefly, cells were harvested and centrifuged at 500 x g at 4℃ for 5 min, then resuspended in hypotonic buffer for 5 min (20 mM Tris-HCl [pH 7.4], 10 mM KCl, 2 mM MgCl_2_, 1 mM EGTA, 0.5 mM DTT). Next, cells were lysed with IGEPAL® CA-630 (I8896-50ML, Sigma) at 0.3% final concentration, and centrifuged at 800 x g to separate the nuclei. Nuclei were resuspended in isotonic buffer (20 mM Tris-HCl [pH 7.4], 150 mM KCl, 2 mM MgCl_2_, 1 mM EGTA, 0.5 mM DTT, 0.3% NP-40) and incubated for 10 min. Finally, nuclei were centrifuged at 800 x g, then washed and resuspended with PBS.

The cells and nuclei were stained with DAPI (C1002, Beyotime Biotechnology, Shanghai, China), ER-Tracker Green (C1042S, Beyotime), and Mito-Tracker Red CMXRos (C1049B-50μg, Beyotime), according to the manufacturer’s instructions. The microscope slides were analyzed using a Nikon A1R confocal microscope (100X).

The quality of the extracted nuclei was also evaluated by quantifying the expression levels of glyceraldehyde-3-phosphate dehydrogenase (GAPDH) and ribosomal protein L30 (RPL30) mRNAs. Total RNA from cells and nuclei was extracted using Direct-zol RNA Microprep Kit (R2060, Zymo Research Corporation), and treated with gDNA wipe mix at 42 °C for 2 min. Reverse transcription of the treated RNA was performed using the HiScript II Q Select RT SuperMix for qPCR (R233-01, Nanjing Vazyme Biotech Co., Ltd). RT-qPCR was performed using AceQ qPCR SYBR Green Master Mix (Q111-02, Vazyme) on an Applied Biosystems 7500 Fast Real-Time PCR System. The data were normalized to the levels of U6 snRNA levels which were stably expressed.

### Human lung cancer specimen preparation

All the included patients were pathologically diagnosed with lung adenocarcinoma. About 1cm^3^ of tumor tissue and tumor adjacent tissue during curative surgery were collected and dissociated into single-cell suspensions by Tumor Dissociation Kit (130-096-730, Miltenyi). Cell suspensions were added with ACK lysis buffer (A10492-01, Gibco) to lyse the remnant RBC, and treated with Dead Cell Removal Kit (130-090-101, Miltenyi), then filtered through the 40-μm plastic mesh (Falcon). Cell suspensions were stained with DAPI for viability, and living cells were sorted with a BD FACSAria III instrument.

### PSCSR-seq library preparation and sequencing

The schema of the PSCSR-seq method is illustrated in Fig. 1.

#### Cell staining and selection

Cell suspensions were stained with ReadyProbes® Cell Viability Imaging Kit (R37610, Thermo Fisher Scientific) for 20 min, then centrifuged (300 x g, 5 min) and resuspended in 1 x DPBS. After cell counting, the stained cell suspensions were diluted in a mix of 1 x Second Diluent (640196, TaKaRa Bio USA) and 0.4 U Ribonuclease Inhibitor (N2515, Promega) to 1 cell/35 nl. The cell suspensions were dispensed into a SMARTer ICELL8 350v Chip (640019, TaKaRa Bio USA, containing 5,184 [72 × 72] nanowells) on a MultiSample NanoDispenser (MSND, TaKaRa Bio USA). All nanowells of the ICELL8 chip were imaged with a fluorescence microscope (Olympus BX43), and the images were analyzed using CellSelect software (TaKaRa Bio USA) to determine the viability and number of cells present. Alive single cells were automatically or manually selected for the subsequent experiments.

#### Small RNA ligation on chip

After single-cell sorting, lysis buffer (35 nl) with 0.5% Triton™ X-100 (T9284, Sigma-Aldrich) and 4 U/μl Ribonuclease Inhibitor was dispensed into selected nanowells. The microchip was transferred to a modified SmartChip thermocycler (Bio-Rad) at 25°C for 5 min and 75°C for 10 min and chilled on ice immediately. 3'-ligation mix (35 nl) containing 0.07 pmol 3' adapter (RA3-A2N, Supplementary Table S3), 60 U/μl T4 RNA Ligase 2, truncated KQ (M0373S, NEB), 3x T4 RNA ligase buffer, and 1.5 U/μl Ribonuclease Inhibitor was prepared and dispensed into the previously selected nanowells. The microchip was incubated with a program of 25°C for 6 hours and 4°C for 8–10 hours, followed by 65°C for 20 min. After 3'-ligation, 35 nl of RT primer mix (0.7 pmol barcoded RT primer [SCSR-RTP, Supplementary Table S3], 2.5 x lambda exonuclease buffer) was added into selected nanowells, with the program “Index 1” on the MSND. The chip was placed inside a thermocycler at 70°C for 2 min and then chilled on ice. 35 nl of adapter-removed solution (2.5 U/μl Lambda exonuclease [EN0562, Thermo Fisher Scientific], 5 U/μl 5' Deadenylase [M0331, NEB], 2.5 U/μl Ribonuclease Inhibitor) was dispensed into the microchip, and incubated at 30°C for 30 min followed by 37°C for 60 min and then 75°C for 10 min. Next, 35 nl of 5'-ligation mix (0.15 pmol of the 5' adapter [SR5F, Supplementary Table S3], 6 mM ATP, 9 U/μl T4 RNA Ligase 1 [M0437M, NEB], 3 x T4 RNA ligase buffer, 3 U/μl Ribonuclease Inhibitor) was added into the microchip and transferred to the thermocycler with a program of 37°C for 60 min and 65°C for 20 min.

#### Reverse transcription and barcoding on chip

The reverse transcription reaction mix (0.9 x first-strand buffer, 50 mM DTT, 1.4 mM dNTP, 2 U/μl ribonuclease inhibitor, 28 U/μl Superscript III reverse transcriptase [18080-085, Thermo Fisher Scientific]) was dispensed into selected nanowells (35 nl of each) and incubated at 55°C for 50 min and 70°C for 15 min. 35 nl of PCR-1 mix (0.3 pmol barcoded PCR-1 primers [SR5F-P1, Supplementary Table S3], 0.8 mM dNTP, 3.2 x PCR buffer, and 0.16 U/μl Phanta® HS Super-Fidelity DNA Polymerase [P502-d1, Vazyme]) was dispensed into the microchip with the program “Index 2”. After dispensation, the microchip was placed in the thermocycler with a program of 95°C for 3 min, followed by 12–14 cycles (95°C for 20 s, 65°C for 20 s and 72°C for 20 s) and a final incubation at 72°C for 5 min. Finally, the microchip was inverted and centrifuged at 3000 x g for 10 min to collect and pool all contents into a single collection tube using the supplied SMARTer™ ICELL8® Collection Kit (640048, TaKaRa Bio USA).

#### Purification and size selection

The collected PCR-1 product was purified using 1.7 x Ampure XP beads (A63882, Beckman Coulter). The size distribution was obtained with an Agilent High Sensitivity DNA Kit (5067-4626, Agilent Technologies) on an Agilent Bioanalyzer 2100 instrument. The quantification was performed with a Qubit™ dsDNA HS Assay Kit (Q32854, Thermo Fisher Scientific). The PCR-1 product was size selected with 3% agarose, dye-free, Pippin Prep (CDP3010, Sage Science) at 125–160 bp.

#### Library amplification and sequencing

The 50 µl PCR-2 reaction mix was prepared with purified PCR-1 product, 0.2 mM dNTP, 1 x PCR buffer, 0.02 U/μl Phanta® Max Super-Fidelity DNA Polymerase (P505-d1, Vazyme), 0.2 µM SCSR-PCR1 primer (Supplementary Table S3), and 0.2 µM SCSR-PCR2 index primer (Supplementary Table S3). The reaction was performed with a program of 95°C for 3 min, 7–13 cycles of 95°C for 20 s, 67°C for 20 s, and 72°C for 20 s, and 1 cycle of 72°C for 5 min. The PCR-2 product was purified with 1.7 x Ampure XP beads. The PSCSR-seq library was quantified with a qPCR-based KAPA Library Quantification Kit for Illumina platforms (KK4824, Kapa Biosystems). The PSCSR-seq library was sequenced using an Illumina HiSeq2500 or NextSeq2000 instrument.

### PSCSR-seq data analysis

#### Sequence analysis

The analytical procedures from our previous publication were used^43^. Briefly, the small RNA sequences, cell barcodes and molecular UMI counts were extracted from the raw reads using custom scripts. The small RNA sequences with a length of 16-39 nt were retained and mapped to the reference genome, with no mismatches allowed (Bowtie V1.2.2^44^). The small RNAs were annotated according to information within the miRbase (release v22.1^45^), EBI RNAcenter (release v11^46^), and EBML CDS databases (ENSEMBL release v96^47^). The computational pipeline can be found within GitHub repository (https://github.com/biocaitao/PSCSR-seq).

#### Cell selection

Viable single cells in nanowells were selected using the ICELL8 system protocol (“StandardCellSettings-V5” or “PBMCv4”). The cell barcodes of the selected cells were then exported. The valid cells were filtered according to the exported barcodes (the length of cell encoded barcodes is 12 nt and barcodes design can tolerate 2-base sequencing error).

#### Saturation analysis

$${\text{Saturation}} = 1 - \frac{{{\text{N}}_{{{\text{deduped}}\_{\text{reads}}}} }}{{{\text{N}}_{{{\text{total}}\_{\text{reads}}}} }},$$

where N_deduped_reads_ = total UMI counts in the cell. N_total_reads_ = total mapped sRNA read counts in the cell.

#### Unsupervised cluster analysis

For each annotated small RNA, reads with same index tag or adjacent index tag (1 mismatch) were collapsed into UMI. After that, the UMI counts were weighted by the number of mapped locations. The UMIs were summed as the measurement of small RNA expression. Small RNA expression values for each cell were normalized and log transformed. Unsupervised cluster analysis was performed as described in a previous study^7^, and the cluster analysis was implemented using the "Seurat" package (v3.0.0)^48^. First, informative small RNA-encoding genes were selected based on the “variable stable transform” algorithm. The small RNA expression profiles were then scaled and projected to the PCA space. The first 50 PCs were selected. The scaled expression profiles were clustered using a graph-based clustering algorithm (the “Louvain” algorithm in Seurat). The resolution parameters were checked from 0.1 to 1 (in 0.1 steps), and we chose a resolution of 0.5 for the data presentation and comparison. The highly expressed genes in each cluster were identified using the criteria of a corrected P value<0.01 (the P values were assessed by the Wilcoxon rank sum test) and a minimal fraction in the population>0.2, log-transformed fold change > 1; other parameters used the default setting of the Seurat “FindAllMarkers” function.

#### Cell ontology analysis

The miRNA expression profiles with cell ontology annotations were downloaded from the FANTOM5^18^ website. Spearman correlations between the new samples and the annotated samples were calculated. For each cell ontology term, the average correlation coefficients between members in the category and those that are not members were then compared. P values were assessed using a modified t-test^49^.

$${\text{t}} = \left( {{\text{m}} - {\text{M}}} \right)/\sqrt {\frac{{{\text{s}}^{2} }}{{\text{n}}} + \frac{{{\text{S}}^{2} }}{{\text{n}}}}$$

$${\text{df}} = \left( {{\text{n}} - 1} \right)\frac{{\left( {{\text{s}}^{2} + {\text{S}}^{2} } \right)^{2} }}{{{\text{s}}^{4} + {\text{S}}^{4} }}$$

Here, m, s, and n are the mean Spearman coefficient, standard deviation, and the number of samples associated with a particular cell ontology term, respectively, and M and S are the mean Spearman coefficients and standard deviations for all of the samples in the dataset.

#### Comparison of methods

Raw reads from existing methods were downloaded. The adapters were removed according to the following patterns:

“HHHHHHHHCA” + sRNA + “TGGAATTCTC” (H for A,C,T; Faridani method and Hücker method);

sRNA + "TGGAATTCTC" (Wang method and Bulk-seq);

“AAANNAAANNAAANN” + sRNA + “NNCTGTAGGCAC” (N for A,C,G,T; PSCSR-seq);

The non-small RNA reads were removed by size filtering (<16nt or >39nt after removal of adapters), and then the small RNA reads were mapped to the genome. The perfectly mapped small RNA reads (allowing no mismatch) were counted.

The small RNA ratios were calculated as below:

For each cell in PSCSR-seq,

$$\frac{{\text{small RNA reads number of the cell}}}{{\text{Total reads number of the cell}}}*\frac{{\text{Total reads number with cell barcodes}}}{{\text{Total sequenced reads number}}}$$

For each cell/sample in other methods,

$${\text{Small}}\;{\text{RNA}}\;{\text{ratio}} = \frac{{{\text{small}}\;{\text{RNA}}\;{\text{reads}}\;{\text{number}}}}{{{\text{Total}}\;{\text{sequenced}}\;{\text{reads}}\;{\text{number}}}}$$

For the reproducibility comparison in Fig. 1E, we followed the definition in the paper of Wu, A.R. et al, 2014^50^.

$${\text{Reproducibility}} = \frac{{{\text{Intersect}}\left( {{\text{small}}\;{\text{RNA}}\;{\text{set}}\;{\text{in}}\;{\text{cell}}1,\;{\text{small}}\;{\text{RNA}}\;{\text{set}}\;{\text{in}}\;{\text{cell}}2} \right)}}{{{\text{Union}}\left( {{\text{small}}\;{\text{RNA}}\;{\text{set}}\;{\text{in}}\;{\text{cell}}1,\;{\text{small}}\;{\text{RNA}}\;{\text{set}}\;{\text{in}}\;{\text{cell}}2} \right)}}$$

Pairwise comparisons among cells were calculated, and the values were averaged.

For the sensitivity comparison in Fig. 1F, the reads with correct adapter sequencing (Hücker method) or cell barcodes (PSCSR-seq) were randomly sampled. Then, the extracted small RNA reads were mapped to the genome, and the annotated miRNAs were counted.

The demo codes can be found within the GitHub repository (https://github.com/biocaitao/PSCSR-seq).

### Ethics declarations

All patients were provided informed consent for sample collection and data analysis. The study was approved by the Ethics Committees of the National Institute of Biological Sciences, Beijing, and of Beijing Chao-Yang hospital of Capital Medical University. We complied with all relevant ethical guidelines and regulations.

## Supplementary Information

Supplementary Information.

Supplementary Figures.

Supplementary Table 1.

Supplementary Table 2.

Supplementary Table 3.

Supplementary Table 4.

Supplementary Table 5.

## Data Availability

The datasets supporting the conclusions of this article are available in the NCBI Gene Expression Omnibus (GEO; https://www.ncbi.nlm.nih.gov/geo/) database under accession number GSE134004. Single-cell miRNA profiles from lung cancer can be visualized through the UCSC cell browser^51^ (https://biocaitao.github.io/lungcancer/). The datasets for methods comparison in Fig. 1D were downloaded from the NCBI GEO database under accession numbers: GSM2149190-GSM2149206 for the Faridani method, GSM3132059-GSM3132078 for the Wang method, GSM5360035-GSM5360040 for Hücker method, and GSM5897090, GSM5897091, GSM5897092, GSM5897094, GSM5897096, GSM5897100 for traditional bulk small RNA sequencing method.

## Code availability

All source codes are available within the GitHub repository (https://github.com/biocaitao/PSCSR-seq).
