# Supplementary material for: Small RNA transcriptome analysis using parallel single-cell small RNA sequencing
Source: Sci Rep. 2023 May 9;13:7501. doi: 10.1038/s41598-023-34390-7 (PMC10170110; doi:10.1038/s41598-023-34390-7)
Supplement: Supplementary file 1 — Supplementary Information. [file 41598_2023_34390_MOESM1_ESM.docx]

**Supplementary Information for**

Small RNA transcriptome analysis using parallel single-cell small RNA sequencing

Jia Li, Zhirong Zhang, Yinghua Zhuang, Fengchao Wang, Tao Cai*

*Corresponding author.

Email: caitao@nibs.ac.cn

**This PDF file includes:**

Supplementary text

Figures S1 to S10

Tables S1 to S5

Supplementary Information Text

**Total RNA extraction and quantification**

Total RNA, including small RNAs (17-200 nt) was isolated using a miRNeasy Mini Kit (217004, Qiagen) following the manufacturer’s instructions. The quantity of total RNA was determined by a Qubit RNA assay kit (Q32852, Thermo Fisher Scientific), and the quality was checked using an Agilent RNA 6000 Nano Kit (5067-1511, Agilent Technologies).

**Small RNA library preparation of bulk cells, total RNA, and synthetic miRNAs**

A total of 1 μl cell suspensions was added in 2 μl of lysis buffer (0.5% Triton™ X-100 and 2 U/μl Ribonuclease Inhibitor), incubated at 25°C for 5 min and 75°C for 10 min, chilled on ice immediately. 1 μl of RA3-A2N adapter (3 pmol) was added and incubated at 70°C for 2 min, then 2 μl of 3’-ligation mix (50 U/μl T4 RNA Ligase 2, truncated KQ, 3x T4 RNA ligase buffer, 3 U/μl Ribonuclease Inhibitor) was transferred, and incubated at 25°C for 60 min and 65°C for 20 min. 1 μl of RT primer(15 pmol) was added and incubated at 70°C for 2 min, then 3 μl of adapter-removed solution (2x Lambda Exonuclease buffer, 1.7 U/μl Lambda Exonuclease, 8.3 U/μl 5’ Deadenylase, 2 U/μl Ribonuclease Inhibitor) was added, and incubated at 30°C for 30 min, 37°C for 60 min and 75°C for 10 min. Afterward, 5 μl of 5’-ligation reaction (5 pmol SR5F adapter, 3 mM ATP, 6 U/μl T4 RNA Ligase 1, 1x T4 RNA ligase buffer, 1.5 U/μl Ribonuclease Inhibitor) was added to the tube with a program of 37°C for 60 min and 65°C for 20 min. 5 μl of Reverse transcription mix (1x First-strand buffer, 40 mM DTT, 2 mM dNTP, 2 U/μl Ribonuclease Inhibitor, 20 U/μl Superscript III reverse transcriptase ) was added and incubated at 55°C for 50 min, 70°C for 15 min. The PCR-1 amplification was carried out by adding 20 μl of PCR-1 mix (10 pmol barcoded SR5F-P1, 0.5 mM dNTP, 2x PCR buffer, 0.04 U/μl Phanta® HS Super-Fidelity DNA Polymerase), with a program of 95°C for 3 min, followed by 10-12 cycles (95°C for 20 s, 65°C for 20 s and 72°C for 20 s) and a final incubation at 72°C for 5 min. After being purified with Ampure XP beads and size selected with Pippin prep, PCR-1 product was amplified with 2x PCR-2 mix (0.5 mM dNTP, 2x PCR buffer, 10 pmol SCSR-PCR1 primer, 10 pmol SCSR-PCR2 index primer, and 0.04 U/μl Phanta® Max Super-Fidelity DNA Polymerase), with a program of 95°C for 3 min, 8-12 cycles of 95°C for 20 s, 67°C for 20 s, and 72°C for 20 s, and 1 cycle of 72°C for 5 min. Total RNA and Synthetic miRNA libraries were generated similarly as described above, starting with 3’ adapter ligation. The sequences of related adapters can be found in Table S3.

Raw small RNA reads were counted as expression values and the statistical comparisons were conducted using the “edgeR” package of R language.

**Microinjection ligation experiment**

Microinjection holes of 200 μm diameter were drilled on a cell culture dish (430164, corning), and the dish was filled with mineral oil (M8410-1L, Sigma-Aldrich). A lysis-ligation mix of Triton™ X-100, Ribonuclease Inhibitor, 3’ adapter, T4 RNA Ligase 2, truncated KQ, and T4 RNA ligase buffer, was injected into the microinjection holes using Olympus Inverted Microscopes (IX71, Olympus), forming ~100 μm diameter ligation-droplets. 10 cells were hand-picked randomly under the microscope, and injected into a ligation droplet, forming a ~150 μm diameter reaction droplet. Similarly, 1 pg of synthetic miRNA(std2) and 100 pg of Universal Human miRNA Reference RNA (750700, Agilent) were separately injected into ligation droplets as well. All the samples had three repetitions. After being incubated at 25°C for 10 hours, the microinjection reaction droplets were moved into PCR tubes for subsequent experiments following the protocol of “small RNA library preparation of bulk cells, total RNA, and synthetic miRNAs”.

According to the calculation formula of spherical volume:

$$V=\frac{4}{3}{\pi r}^{3}$$

The volume of the reaction droplet was ~2 nl. As a control, libraries with the same start materials were prepared in PCR tubes, except the volume of the ligation reaction was 5 μl (see Supplementary Fig.S1G).

Fig. S1. Nanoliter-scale reactions improve the product yield of small RNA libraries

(**A-C**): Electropherograms (with an Agilent Bioanalyzer 2100) of small RNA libraries using a tube method (microliter-scale reaction), starting with (**A**) 1 pg of synthetic miRNAs, (**B**) 100 pg of Universal Human miRNA Reference RNA (750700, Agilent), and (**C**) a pool of 10 A549 cells; (**D-F**): corresponding microinjection method (nanoliter-scale reaction, see the “microinjection ligation experiment” section of Supplementary Text), (**D**) 1 pg of synthetic miRNAs, (**E**) 100 pg of Universal Human miRNA Reference RNA, and (**F**) a pool of 10 A549 cells; (**G**) microinjection operation using a microscope. (**H**) the number of detected miRNAs using Universal Human miRNA reference RNA (n=3, error bars for standard deviation, 10M reads were sampled for the analysis).

Fig. S2. The product yield of small RNA libraries under different conditions

Electropherograms of small RNA libraries from A549 cells (see the “small RNA library preparation of bulk cells, total RNA, and synthetic miRNAs” section of Supplementary Text); After adding lysis buffer, samples were heated for 10 min at 25°C (**A**), 37°C (**B**), 75°C (**C**), 80°C (**D**), 85°C (**E**), 95°C (**F**) and for 5 min at 70°C (**G**), and 75°C (**H**).

Fig. S3. The high sensitivity of the PSCSR-seq design

Sensitivity comparison between the PSCSR-seq protocol (optimized adapters, “N” samples) and the standard protocol (Illumina protocol, “S” samples) using synthetic miRNA oligos.

Boxplot for the normalized abundance of synthetic miRNA oligos (miRNA expression values were divided by the median value and log10 transformed. The normalized values were expected to be 0 after log10 transformed). The miRXplore Universal Reference samples (Miltenyi Biotec, a total of 962 different miRNAs) were used for the analysis with input: (**A**) 0.1ng, (**B**) 1ng, and (**C**) 10ng.

4M reads from each sequencing file were sampled for comparisons. Pvalues were assessed by the Kolmogorov–Smirnov test, and the detected miRNA numbers were highlighted in the plot.

Fig. S4. The high-quality single-cell small RNA sequencing with PSCSR-seq

(**A**) Electropherograms of a small RNA library of PSCSR-seq (top), the adapter-only negative control (middle), and the small RNA library after the size selection step (bottom). (**B**) the average reads distribution of different methods, see the “comparison of methods” section of Methods.

Fig. S5. The high reproducibility of PSCSR-seq

(**A**) Scatter plot of small RNA expression profiles between two A549 cells; each circle represents one annotated small RNA; (**B**) scatter plot of average small RNA expression profiles between nanowell chips (technical replicates), the reproducibility was defined in the “comparison of methods” section of Methods.

Fig. S6. PSCSR-seq analysis of cultured cells

(**A**) Cell selection plot.

UMI counts for 5,184 nanowells are sorted and plotted. The black points indicate selected wells (wells with a living cell), the grey points indicate background wells, and the red line indicates the cutoff for high-quality data (inflection point of the curve).

(**B**) Saturation curve for A549 cells.

The estimated saturation at different sequencing depths is plotted (see the "saturation analysis" section of Methods).

(**C**) Histogram of the number of most abundant miRNAs contributing 50% of the total miRNA expression in each cell.

(**D**) Comparison between PSCSR-seq and bulk-seq analysis. The Venn diagrams show the significant overlap of identified cell type marker miRNAs between PSCSR-seq and bulk-seq analysis. P<0.001 for all cell types (Fisher's exact test).

(**E**) Quantile-quantile plots (Q-Qplot) for cell ontology analysis.

The quantiles of the observed t-statistics versus the theoretical quantiles of the standard normal are plotted. The most significant terms are presented.

(**F**) tSNE projection of A549 cells calculated from the expression profiles of small RNA forms from rRNAs, snRNAs, or snoRNAs. The proportion of particular small RNA forms among the total molecules in each cell is overlaid on each tSNE plot.

(**G**) tSNE projection of mixed cells calculated from the expression profiles of small RNA forms from rRNAs, snRNAs, or snoRNAs. In the snRNA and snoRNA plots, cells with little information (snRNA/snoRNA species numbers in a cell less than 20) were removed.

Fig. S7. small RNA characteristics of nuclei

(**A**) Size distribution of small RNA reads in nuclei (293T-N and HeLa-N) and cells (293T-C and HeLa-C); (**B**) Reads coverage of let-7a precursor gene, and (**C**) Reads cumulative distribution across multiple miRNA precursors (n=40).

Fig. S8. PSCSR-seq analysis of nuclei isolated from cultured cells

(**A-B**) Separation of nuclei (HeLa-N) and whole cells (HeLa-C). RT-qPCR validation (A) and microscopy visualization (B) of HeLa cells and HeLa nuclei. The samples were stained with DAPI, ER-tracker, and Mito-tracker.

(**C**) Violin plot for the distribution of small RNA molecule counts and miRNA species numbers in HeLa nuclei (HeLa-N) and whole cells (HeLa-C).

(**D**) Scatter plot comparing miRNA expression levels in HeLa nuclei and whole cells. Nuclei or whole cells from PSCSR-seq were pooled together, and the miRNA expression was averaged. The axis is log10 transformed. Red dots indicate the differentially expressed miRNAs. For source data, see Supplementary Table S5.

Fig. S9. PSCSR-seq analysis of fresh PBMCs

tSNE projection plots showing subpopulations and marker miRNA expression. There are 923 cells with an average of 57 miRNAs detected per cell. The composition based on miRNA expression is as follows: Pan T cells, 553 (59.9%), NK cells, 138 (15.0%), B cells, 19 (2.0%), monocytes, 70 (7.6%), and granulocytes, 143 (15.5%). The hierarchical clustering tree shows the lineage relation of subpopulations. Here cells of subpopulations were pooled together for the hierarchical clustering analysis.

Fig. S10. PSCSR-seq analysis of lung cancer

(**A**) tSNE projection plots indicate the sample origin and tumor (miR-135b)/immune (miR-142) marker miRNA expression in matched tumor tissue (TT) and tumor-adjacent tissue (TAT).

(**B**) Density plots of miR-34a/b/c expression across cell types in TT and TAT. In the plot, the x-axis represents the miRNA expression level, and the y-axis represents the density of cells.

Table S1.

Dataset information from this study.

Table S2.

Spearman correlation analysis between PSCSR-seq miRNA profiles and profiles from miRNA atlas databases.

Table S3.

Adapter and primer sequences for PSCSR-seq.

Table S4.

Demographic information from lung cancer patients.

Table S5.

Average miRNA expression in nuclei and whole cells
