# Supplementary figures and images for: Small RNA transcriptome analysis using parallel single-cell small RNA sequencing
Source: Sci Rep. 2023 May 9;13:7501. doi: 10.1038/s41598-023-34390-7 (PMC10170110; doi:10.1038/s41598-023-34390-7)

Figure S1

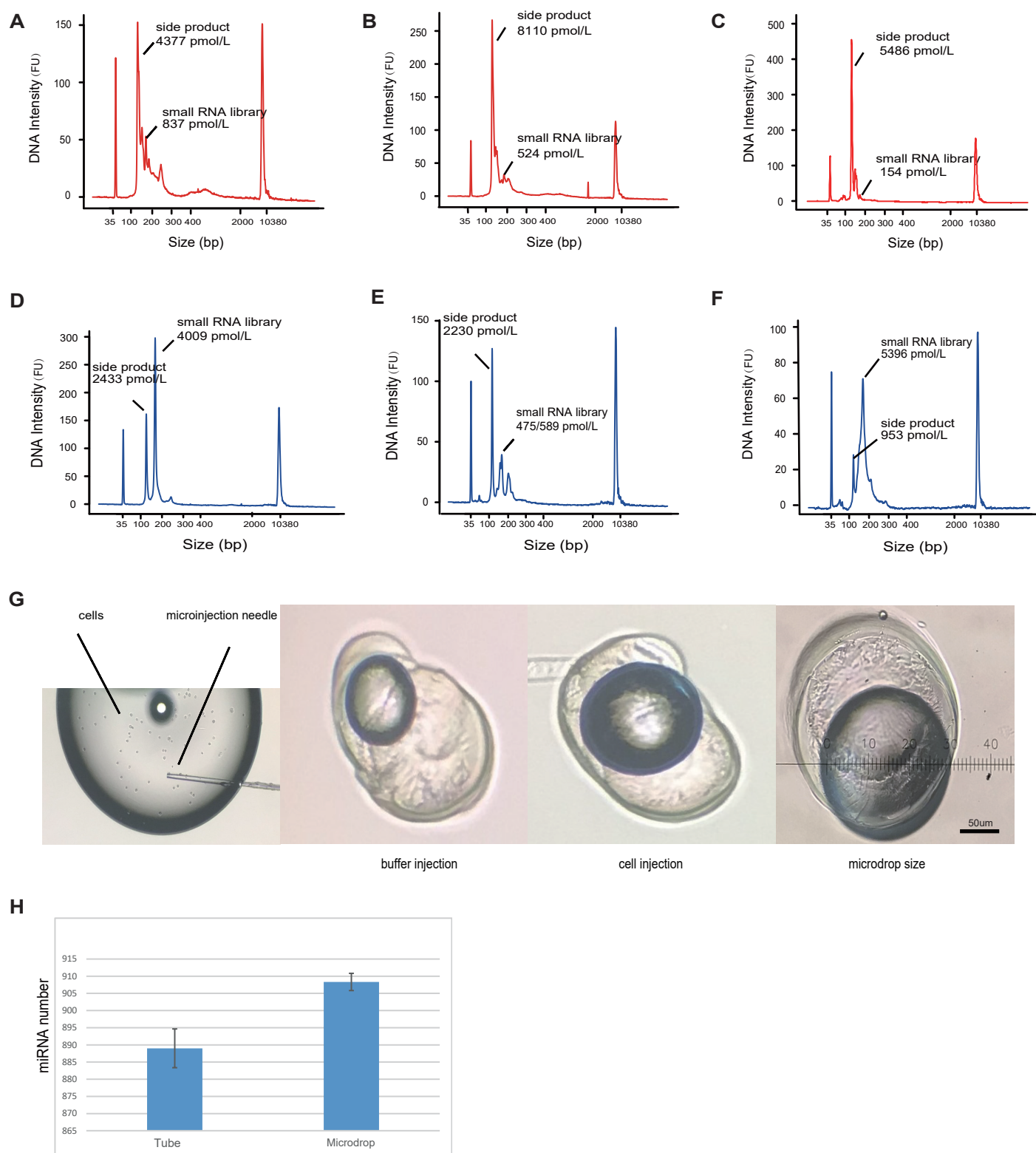

Figure S2

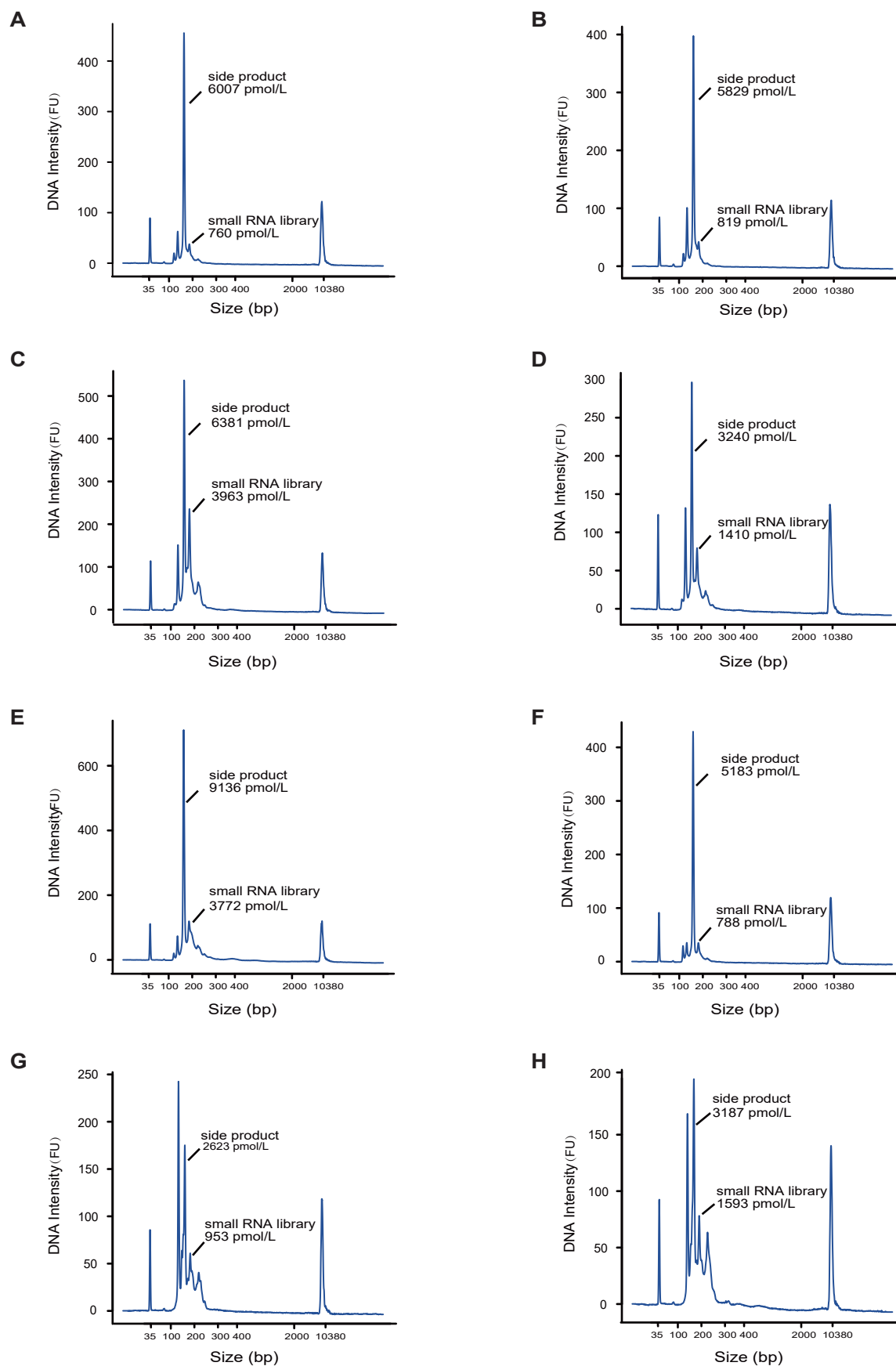

Figure S3

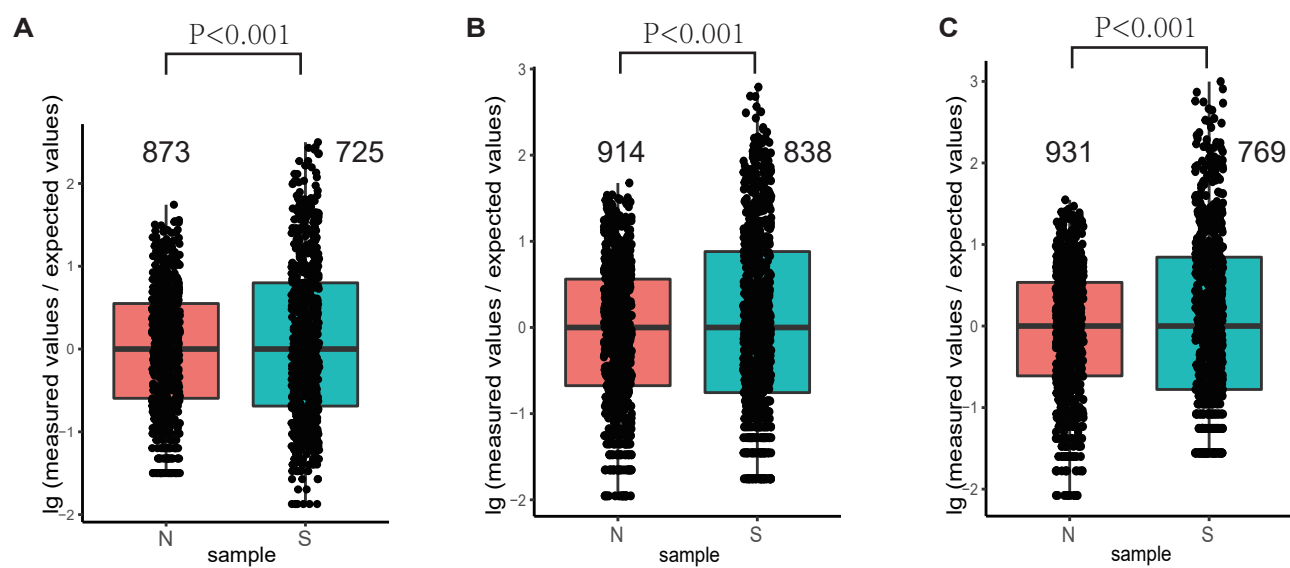

Figure S4

A

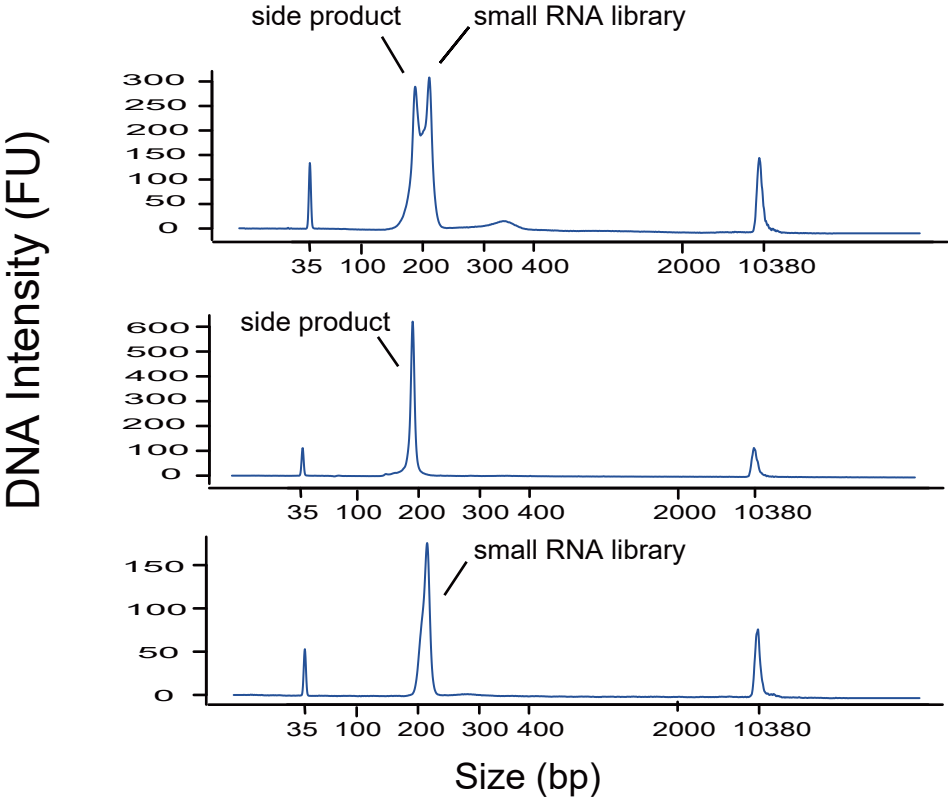

B

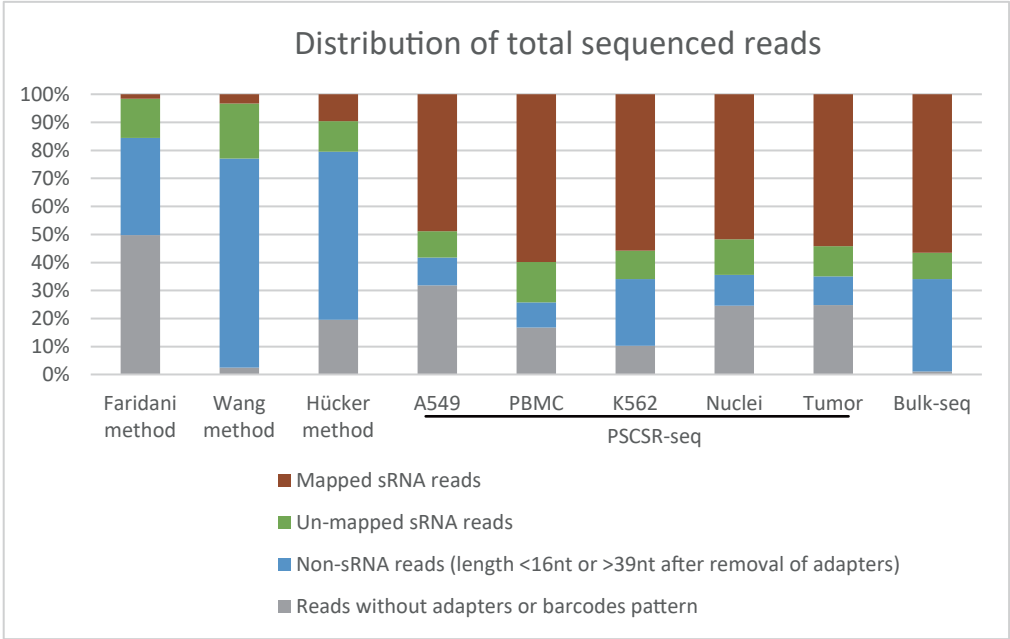

Figure S5

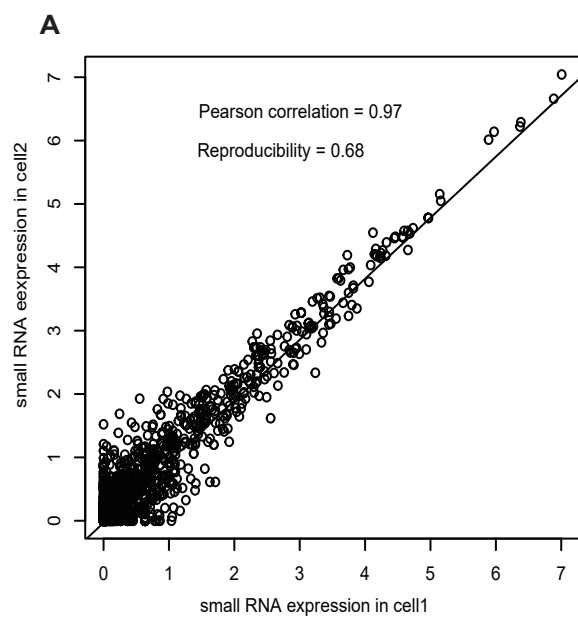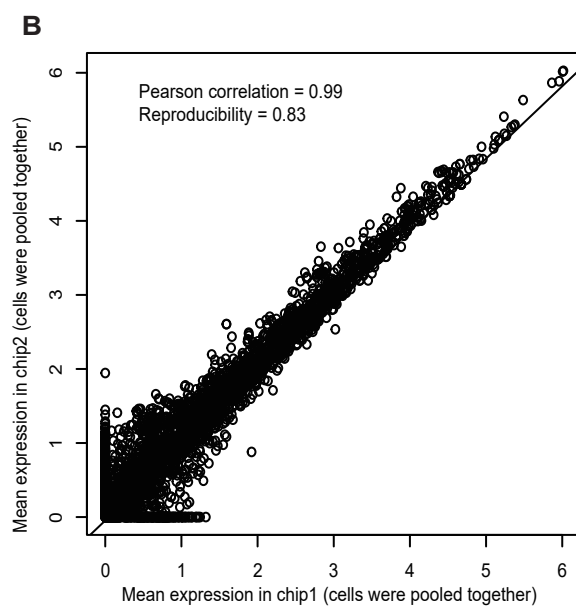

Figure S6

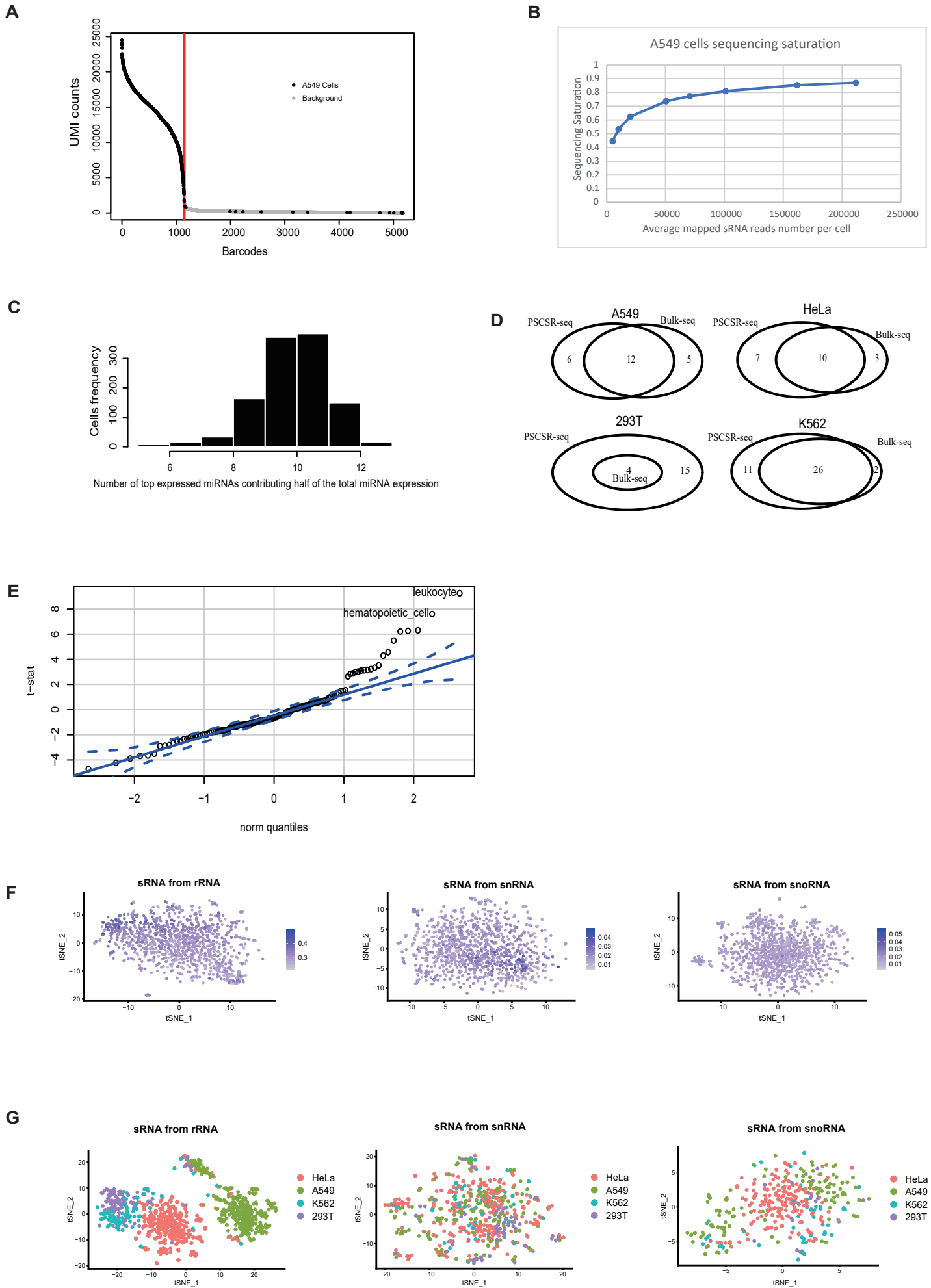

Figure S7

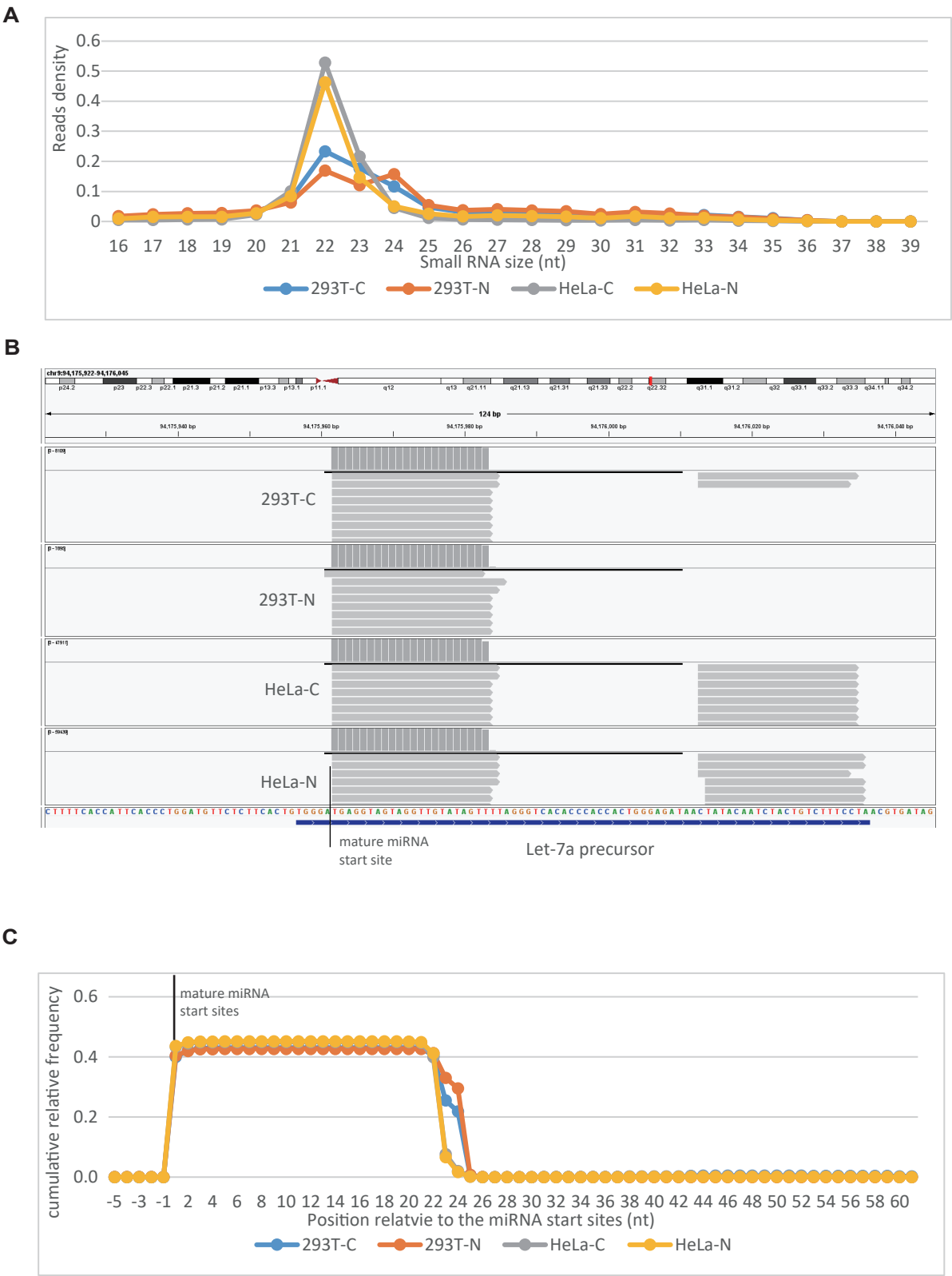

Figure S8

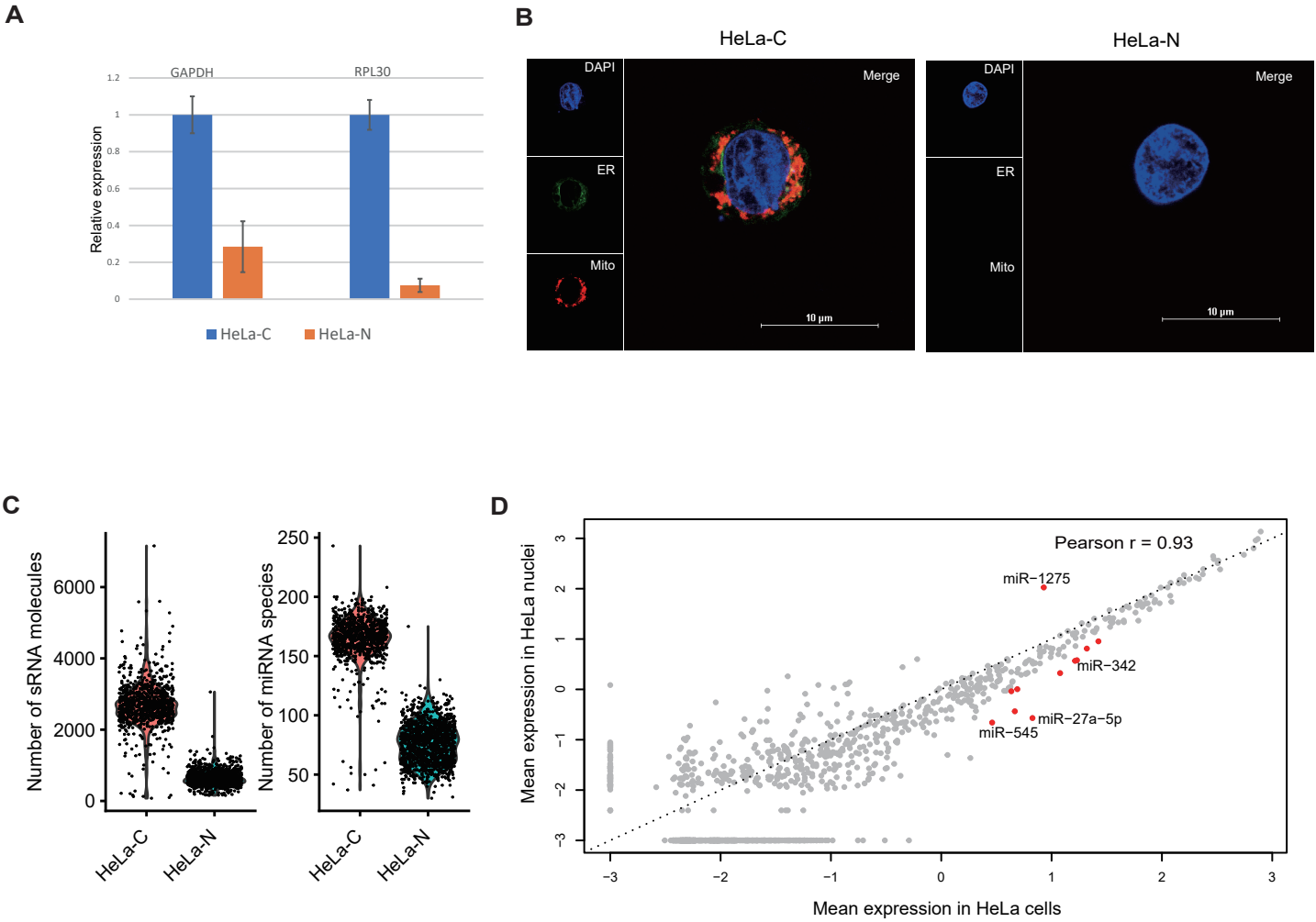

Figure S9

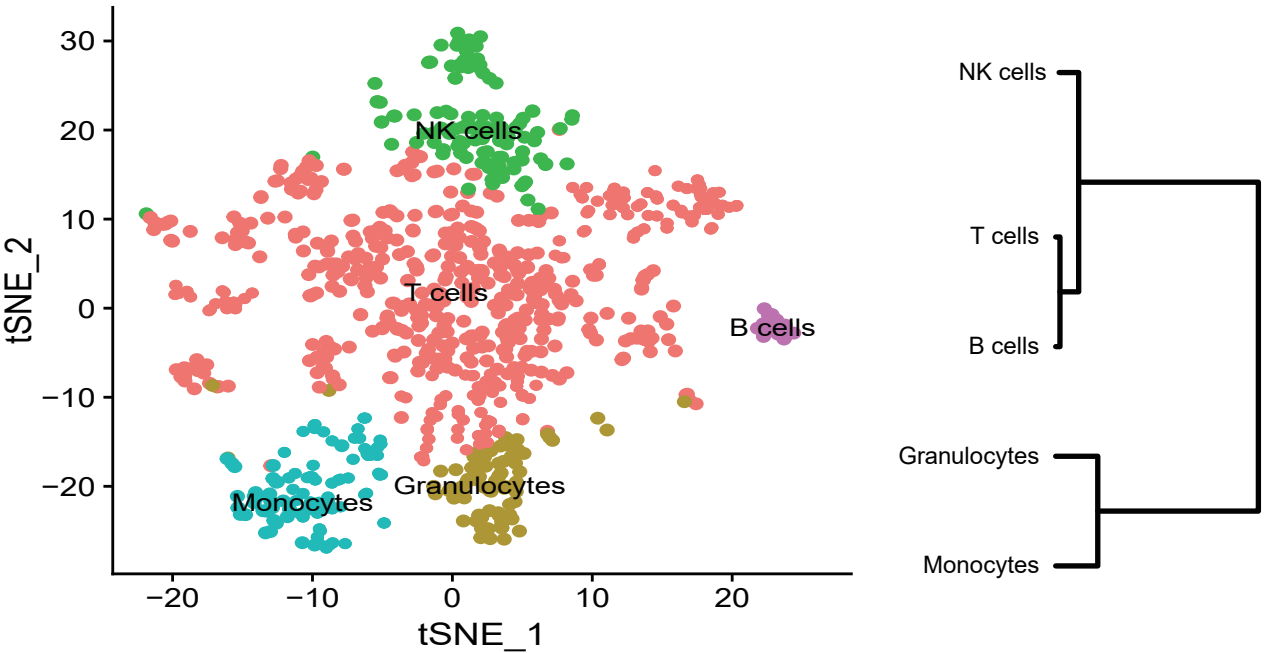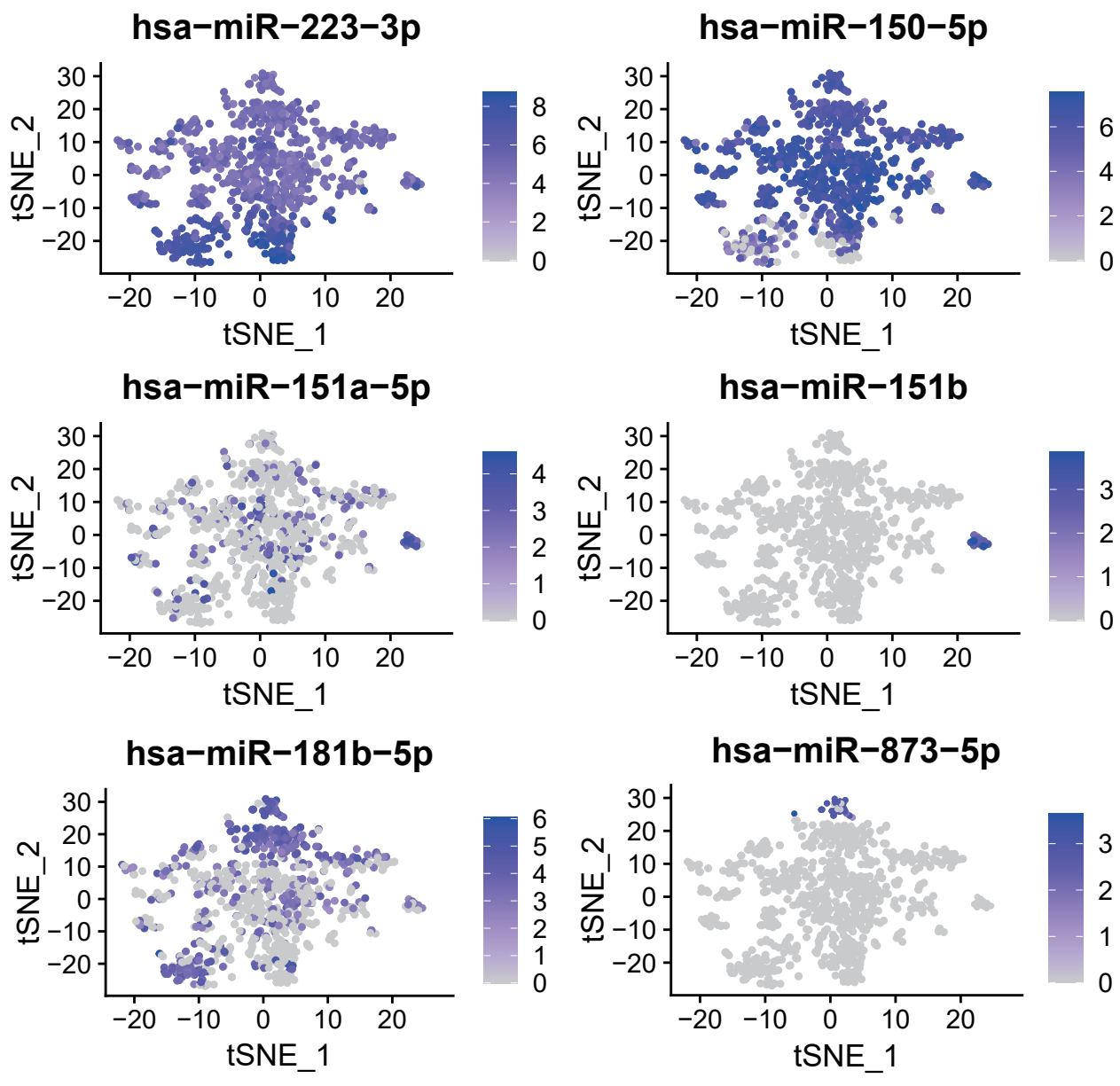

A

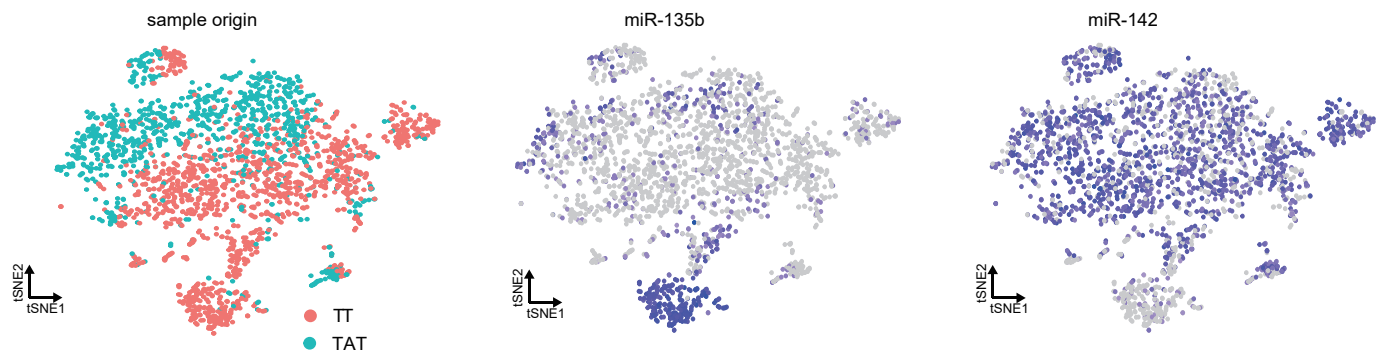

B

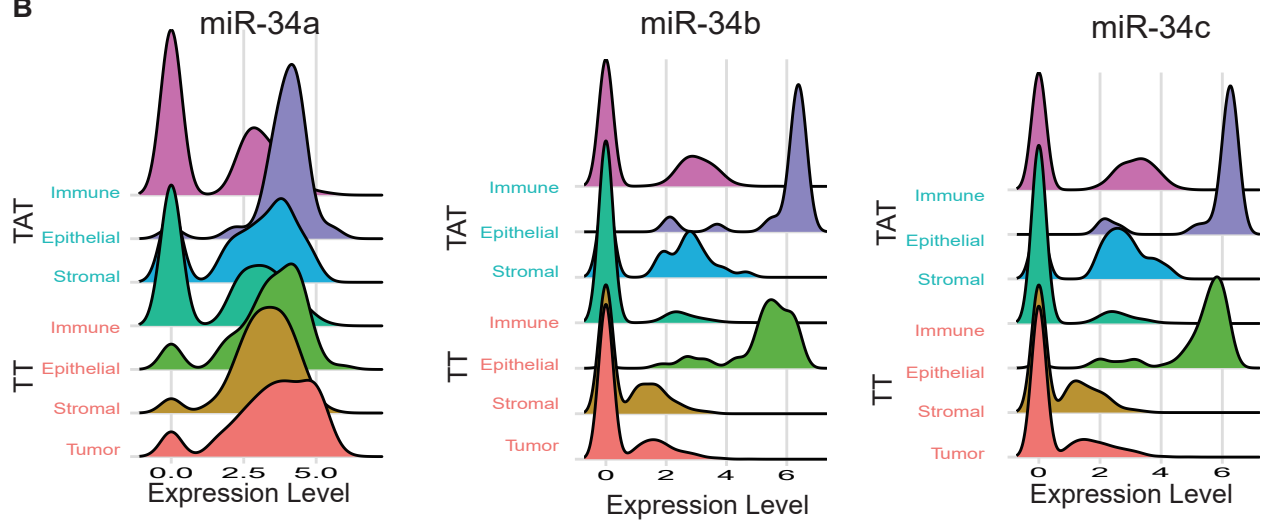

Supplement: Supplementary file 2 — Supplementary Figures. [file 41598_2023_34390_MOESM2_ESM.pdf]
